# First freshwater coralline alga and the role of local features in a major biome transition

**DOI:** 10.1038/srep19642

**Published:** 2016-01-21

**Authors:** A. Žuljević, S. Kaleb, V. Peña, M. Despalatović, I. Cvitković, O. De Clerck, L. Le Gall, A. Falace, F. Vita, Juan C. Braga, B. Antolić

**Affiliations:** 1 Institute of Oceanography and Fisheries, 21000 Split, Croatia; 2 Department of Life Science, University of Trieste, 34127 Trieste, Italy; 3 BIOCOST Research Group, Departamento de Bioloxía Animal, Bioloxía Vexetal e Ecoloxía, Facultade de Ciencias, Universidade da Coruña, 15071, A Coruña, Spain; 4 Research Group Phycology, Ghent University, 9000 Ghent, Belgium; 5 Equipe Exploration, Espèces et Evolution, Institut de Systématique, Evolution, Biodiversité, UMR 7205 ISYEB CNRS, MNHN, UPMC, EPHE, Muséum national d’Histoire naturelle (MNHN), Sorbonne Universités, F-75005, Paris, France; 6 Departamento de Estratigrafía y Paleontología, Universidad de Granada, Campus Fuentenueva, 18002, Granada, Spain

## Abstract

Coralline red algae are significant components of sea bottom and up to now considered as exclusively marine species. Here we present the first coralline alga from a freshwater environment, found in the Cetina River (Adriatic Sea watershed). The alga is fully adapted to freshwater, as attested by reproductive structures, sporelings, and an inability to survive brackish conditions. Morphological and molecular phylogenetic analyses reveal the species belongs to *Pneophyllum* and is described as *P. cetinaensis* sp. nov. The marine-freshwater transition most probably occurred during the last glaciation. The brackish-water ancestor was preadapted to osmotic stress and rapid changes in water salinity and temperature. The particular characteristics of the karst Cetina River, such as hard water enriched with dissolved calcium carbonate and a pH similar to the marine environment, favoured colonization of the river by a marine species. The upstream advance and dispersal is facilitated by exceptionally pronounced zoochory by freshwater gastropods. *Pneophyllum cetinaensis* defies the paradigm of Corallinales as an exclusively marine group.

Coralline red algae (Corallinophycidae, Rhodophyta) are important components of marine ecosystems. They are ubiquitous from tropical regions to the poles, thriving from the intertidal to the lower boundaries of the euphotic zone^1^,^2^. Impregnated with calcium carbonate, they fill a paramount role as ecosystem bio-constructors, consolidating coral reef and coralligenous structures^3^, developing extensive maerl/rhodolith beds^4^, providing three-dimensional habitats, favouring the development of other benthic organisms^5^, and significantly contributing to carbonate deposition in shallow marine water (MW)^6^,^7^.

Coralline algae are today a topical subject for scientists interested in global change^8^,^9^,^10^,^11^. They are frequently used in paleoenvironmental reconstruction and as a biological datalogger for the reconstruction of past sea levels^12^, water temperature^13^,^14^, surface ocean salinity, and freshwater balance^15^ and, as recently proposed, for seawater pH oscillations^8^.

Within the red algal phylum (Rhodophyta), with ca 7,100 living species, Corallinales are the third most species-rich group, with 725 described living taxa^16^. Although some coralline algae can be found in brackish environments^17^, a truly freshwater (FW) representative has never been reported so far, either as a fossil or as a living species. Like other important marine lineages such as echinoderms, corallines have been considered restricted to marine water, never making the evolutionary step into FW.

Here we present the first record of a strictly FW coralline algae, discovered in the Cetina River (Croatia), a karst river of the Adriatic Sea watershed (Figs 1 and 2), to which it seems to be strictly endemic. We assessed that it pertains to the marine genus *Pneophyllum* by both morphological and molecular phylogenetic analyses. We also discuss the processes which facilitated the rare evolutionary adaptation and tremendous habitat shift of a single algal species in a geologically recent time in light of the specific abiotic and biotic characteristics of the karst Cetina River.

## Results

### Species description

***Pneophyllum cetinaensis*** Kaleb, Žuljević & V. Peña, sp.nov.

Phylum: Rhodophyta

Subclass: Corallinophycidae

Order: Corallinales

Family: Corallinaceae

Etymology: Cetinaensis from Cetina, name of the river where species is found.

Holotype: PC0145164 (Herbier Cryptogamique PC, Muséum National d´Histoire Naturelle, Paris, France, Fig. 2a)

Isotypes: PC0145165, PC0145166 and PC0145167, deposited at the National History Museum in Paris (PC). NHMS000566, NHMS000567, NHMS000568, deposited at the Natural History Museum in Split (NHMS). 600:ZAG;1:BOA, deposited at the Croatian Natural History Museum in Zagreb (CNHM). ZA39846, ZA39848, deposited at the Herbarium Croaticum at the University of Zagreb (ZA).

Date of collection: 27.08.2013.

Type locality: Otok Ljubavi (Island of Love), Cetina River, Croatia (43° 26.180’N - 16° 45.785’E).

### Diagnosis

With the characteristics of *Pneophyllum*, it differs from other species in forming extensive and conspicuous crusts thickened and mostly multi-layered with flattened or curved branches, and in having the pore canal of conceptacles simple, not surrounded by specialised cells. It also differs from any known coralline red algae in its ecology, being confined to a freshwater stream as opposed to the marine environment.

### Description

Thalli are non-geniculate, attached ventrally to the substratum (cobbles, pebbles, gastropods, and plant roots) (Fig. 2) by cell adhesion and forming sometimes extensive crusts up to 60 mm across or more (Fig. 2c,d). The thallus is encrusting or layered, arranged in superimposed flattened or curved fragile branches (Fig. 3c), each up to 145 μm thick. The branches are arranged horizontally and in most cases concentrically, and exhibit a irregular whitish or pale pink margin (Fig. 2b). Living thallus is pink to violet with matt texture (Fig. 2). The thallus is developed from a spore germination disc with an eight-celled central element (Fig. 4).

The pseudoparenchymatous thallus has a dorsiventral organisation and dimerous construction and can be bistratose or multi layered. The ventral region consists in a single layer of squarish cells (10–22 μm long × 6–15 μm in diameter) forming a filament more or less parallel to thallus surface. The peripheral region can consist only of the epithallial cell (3–5 μm × 6–9 μm) or is composed of filaments perpendicular to thallus surface, with one or more rectangular cells (6–14 μm × 7–12 μm) (Fig. 3b,c). The subepithallial initials are elongate ranging from 13 to 24 μm long and 5 to 12 μm in diameter. The thallus surface is *Pneophyllum*-type (SEM) with wide lenticular epithallial cells (3–6 μm long and 6–10 μm wide) (Fig. 3a).

Cells of the same filament are joined by primary pit-connections, whereas cells of adjacent filaments (other than epithallial cells and subepithallial initials) are connected laterally by cell fusions. Secondary pit-connections were not found. Trichocytes are common terminally on erect filaments.

Gametangial thalli monoecious with male and female conceptacles occurring in the same thallus. Male conceptacles are uniporate, flat or slightly protruding above surrounding thallus surface, roof 4–5 cells thick above chamber (30–43 μm), chambers conical 24–28 μm high × 83–88 μm in diameter (Fig. 3e). The floor of the conceptacle 6–8 cells below thallus surface. Pore sometimes fringed but without spout. Female conceptacle are uniporate, slightly raised or hemispherical with domed to elliptical chambers 85–93 μm high × 225–232 μm in diameter (Fig. 3f). The floor of the conceptacle 8 cells below thallus surface. Roof composed of 5–6 cells layers (42–64 μm). Pore simple, without papillae. Tetrasporangial conceptacles are uniporate, hemispherical and protruding, with elliptical chambers (60–77 μm high × 130–182 μm in diameter) and a small columella usually present at the base (Fig. 3d). The roof of the conceptacles is 3–5 layers thick (20–37 μm). The floor of the conceptacle chambers is 4–8 cells below the thallus surface. Tetrasporangia are zonate 100–110 μm × 60–64 μm. Old conceptacles never becoming buried within the thallus.

Morphologically, *P. cetinaensis* can readily be delimited from other *Pneophyllum* species known to occur in the European Atlantic and Mediterranean coasts (the generitype *P. fragile*, *P. confervicola*, *P. limitatum*, *P. lobescens*, *P. subplanum, P. myriocarpum*, and *P. zonale*). *Pneophyllum cetinaensis* can form extensive and conspicuous crusts and is the only species with flattened or curved branches arranged in horizontally oriented layers. Moreover the thalli are thickened, mostly multi layered. Erect filaments are known to occur only in *P. zonale*, *P. myriocarpum* and in *P. fragile*. In *P. zonale*, the pore canal of tetra/bisporangial conceptales is surrounded by free, unicellular filaments while in *P. myriocarpum* the pore canal is surrounded by a hyaline collar. By contrast, *P. fragile* lacks such specialized structures in tetra/bisporangial conceptale pore canals; however, unlike *P. cetinaensis*, the spermatangial conceptacles are provided with a spout.

### Molecular phylogenetic analyses

The phylogenetic analyses inferred from the plastid *psb*A gene resolved the FW coralline species *P. cetinaensis* within the genus *Pneophyllum* with strong support (89%/0.78 for ML and BI, respectively, Fig. 5). In our phylogenetic analyses, *P. cetinaensis* was resolved as a distinct lineage, but its exact position in the genus and the relationships with Atlantic and Mediterranean isolates was not resolved (Fig. 5, Supplementary Table S1).

### Distribution and ecology

**Figure 1:**
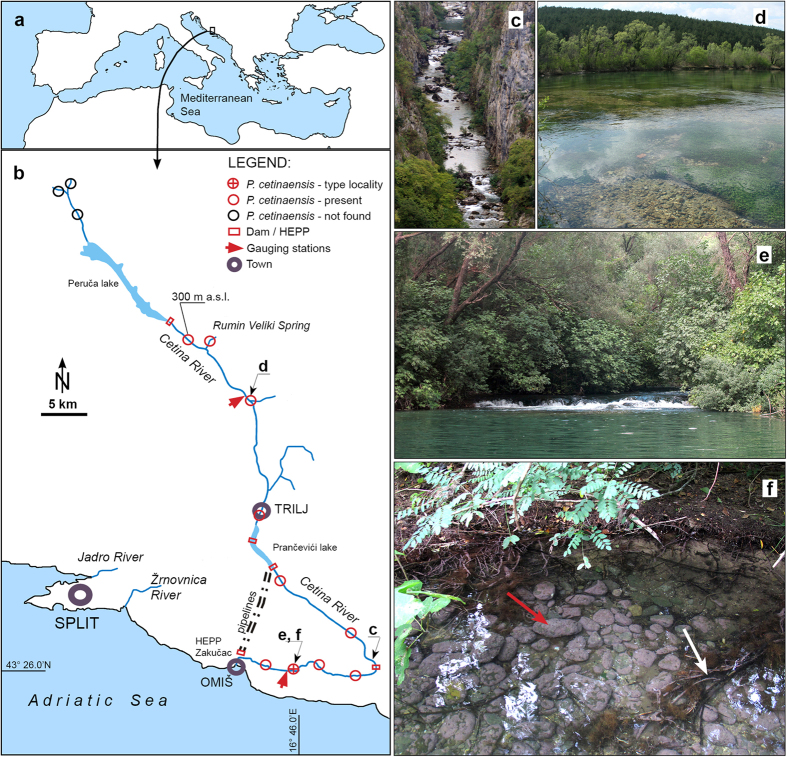
Study area. Cetina River with locations of *Pneophyllum cetinaensis*, including the type locality (**a**,**b**). Different aspects of the river in the canyon (**c**), the plain section (**d**), and the type locality (**e**). *Pneophyllum cetinaensis* in the type locality covers all available hard substrata, such as cobbles and pebbles (red arrow), and roots (white arrow) in shaded, shallow areas of the riverbed (**f**). Maps were created using Adobe In Design CS5 and Photoshop CS5 software and based on OpenStreetMap (https://www.openstreetmap.org/copyright).

*Pneophyllum cetinaensis* is, to the best of our knowledge, present only in the Cetina River (Croatia) and its tributary the Veliki Rumin. Despite intensive searching, the species was not found in the nearby Jadro and Žrnovnica rivers (15 km and 20 km from the mouth of the Cetina River, respectively; Fig. 1).

The Cetina River is a typical permanent karst river of the Adriatic Sea watershed located in a topographically complex karst landscape. It is 105 km long, with the main spring 382 m above sea level and a few short tributaries. The upper course flows across plains, the middle incises a deep canyon, and the lower course runs through a valley. The canyon area has numerous waterfalls, with the 49 m-high Gubavica Waterfall being the highest. Most of the river basin’s bedrock is Cretaceous carbonate rock, mainly limestone. The riverbed ranges from rocky to a cover of cobbles, pebbles, and sand. The river’s water quality is good except in small areas close to towns, where the quality is moderate with a certain degree of pollution^18^. The water can be characterized as hard to very hard (see Table 1 for the basic physicochemical parameters).

The natural hydrological regime of the Cetina River has changed significantly since the 1960s with the construction of hydroelectric power plants (HEPP) and artificial lakes (Fig. 1). Especially in the last 40 km (the type locality), the regime has moderated, showing less seasonal variation^19^. Since 1980, 90% of the water is transported via tunnels from the Prančevići artificial lake to Zakučac HEPP, near the river mouth (Fig. 1). The annual variation in water flow before building this HEPP was a minimum of 20 m^3^ s^−1^ in summer up to 200 m^3^ s^−1^ in winter. Due to water diversion, the mean annual discharge has now dropped from 100 m^3^ s^−1^ to an almost constant 10 m^3^ s^−1^ in the last 40 km of the river course^19^, except for short periods of intense rains. Discharge from the Prančevići artificial lake is estimated to be at least 8 m^3^ s^−1^ to satisfy the ecologically acceptable flow (biological minimum)^19^. At the type locality, a current speed of 20 cm s^−1^ was measured above the pebbled bottom and of 110 cm s^−1^ in moss-covered areas^20^.

**Figure 2:**
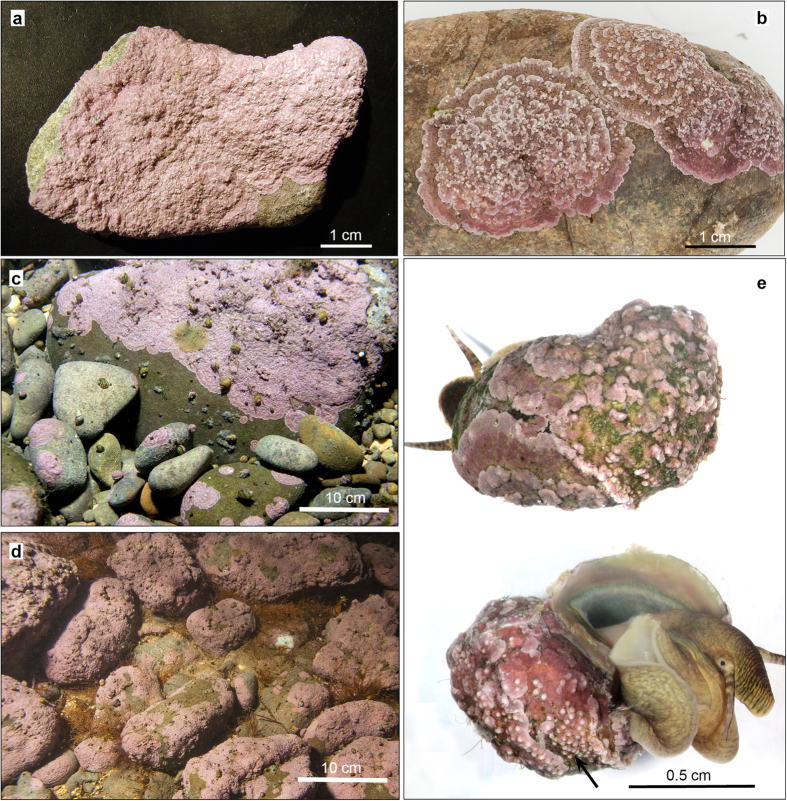
Habitus of *Pneophyllum cetinaensis*. Holotype (PC0145164) (**a**). Typically, *P. cetinaensis* develops as a crust on cobbles and pebbles (**b**,**c**). Extensive coverage in a shaded, shallow area in the type locality (**d**) where most gastropods are overgrown by *P. cetinaensis*, commonly with reproductive structures (arrow) (**e**).

*Pneophyllum cetinaensis* is present throughout almost the entire length of the Cetina River from 0 to 300 m above sea level, which is about 75 km from the river mouth (Fig. 1). The alga was not found upstream of the artificial Peruča Lake. It is probably not present in artificial lakes due to the high variation in water levels. In the estuary, it is found only in the shallowest water layer, down to around 50 cm deep, where there is no seawater influence. *Pneophyllum cetinaensis* develops in areas with either slow or fast water currents from 0 to 2 m deep (Fig. 2). Most commonly, it grows on the self-shaded sides of pebbles and not on substrata directly exposed to the sun. The area with the greatest algal development is the type locality, Otok ljubavi (Figs 1b,f and 2d). On the river margin (0–30 cm deep, 0–1 m from the shore), where water flow is not strong and the bed is shaded by a dense tree canopy (Fig. 1e), the alga nearly completely covers the available hard substratum (mostly cobbles, pebbles, and plant roots) (Figs 1f and 2d), and can be found on most adult gastropods (Fig. 2e). More than 95% of the gastropods collected from cobbles and pebbles are *Theodoxus fluviatilis fluviatilis* (Linnaeus 1758). Algal crusts occurred on 96% of specimens of the latter species larger than 4 mm (n = 267), of which 40% had algae covering more than 50% of the shell surface. In gastropods smaller than 4 mm (n = 124), alga occurred on 39% of gastropods and always covering less than 50% of the shell surface.

## Discussion

Biome transitions are rare macroevolutionary events with profound consequences for terrestrial^21^, FW^22^, and marine habitats^23^. The high diversity of FW species living today is a result of the diversification of a small number of successful ancestral lineages that invaded FW from the marine or terrestrial biomes in ancient geological times^21^.

In spite of the direct contact between MW and FW, invasions of marine species into FW are infrequent events and restricted to a small number of lineages. Of the 31 animal phyla found in the sea, 11 are exclusively marine and have never successfully invaded FW habitats^22^. The rarity of transition and consequences for diversity can be observed in almost every FW animal group. It is estimated, for instance, that just one ancestral lineage of marine sponge has resulted in today’s diversity of around 300 FW sponges^24^. Similarly, less than 40 MW lineages of gastropods have produced today’s total diversity of 4,000 described FW species^25^. Even microbes have only infrequently crossed the marine-FW boundary, and most of those transitions occurred long ago in evolutionary time despite large population sizes, high genetic diversity, and a good potential for long-distance dispersal^26^.

The diversity of FW red algae is surprisingly small compared to MW. There are only about 200 FW species accounting for 3% of red algal diversity^16^,^27^. The majority of FW red alga belong to the order Batrachospermales, which is exclusively found in FW^27^. Other FW species are scattered across the red algal tree of life, classified in species-poor orders mostly confined to FW environments^27^,^28^ (Fig. 6). They are found at very few locations^29^, often surprisingly disjunctive^30^ and in specific, sometimes extreme, environments^31^. Except for two species of the predominantly marine genus *Hildenbrandia*, none of the strictly FW red algae have close relatives in the marine biome, indicating that their ancestral lineages invaded FW in ancient evolutionary time.

There are only 15 species (belonging to the order Ceramiales) which have bridged the MW-FW boundary, but even then they still live in marine habitats where they exclusively reproduce^27^. Ceramiales does not contain a single strictly FW species. Together with FW representatives of *Hildenbrandia*, they can be considered as evolutionary secondary immigrants from the sea^31^,^32^, sharing common characteristics such as the absence of sexual reproduction in FW, while vegetative reproduction is present only by means of gemmae in *Hildenbrandia*^27^.

**Figure 3:**
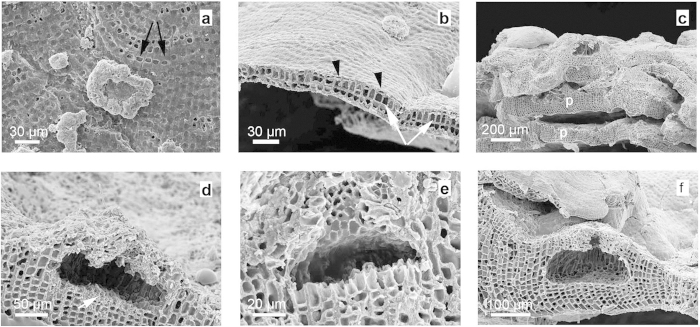
SEM images of the holotype (H) (PC0145164) and the isotype (I) PC0145165. *Pneophyllum*-type surface with lenticular epithallial cells (arrows) (H) (**a**); bistratose thallus with elongate initials (arrows) and one layer of epithallial cells (arrowheads) (H) (**b**); superimposed branches with peripheral region (p) composed of filaments perpendicular to the thallus surface (I) (**c**); sporangial conceptacle with small columella at the base of the chamber (arrow) (I) (**d**); spermatangial conceptacle showing a conical chamber (H) (**e**); female conceptacle showing an elliptical chamber (H) (**f**).

**Figure 4:**
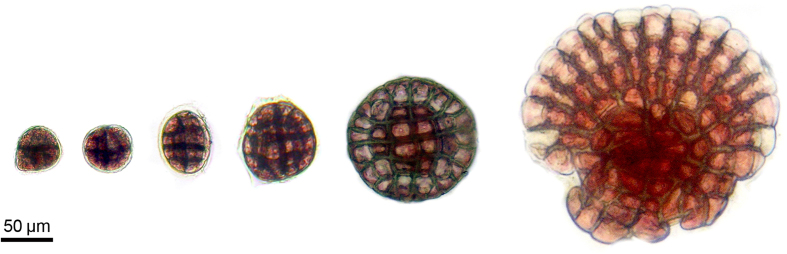
Spore segmentation. Development of the germination disc with an eight-celled central element.

*Pneophyllum cetinaensis,* which we discovered to be strictly endemic to the Cetina River (Croatia) (Figs 1 and 2), is the first known FW coralline alga. It is thus a member of a widely distributed, highly species-rich and diversified order that is immensely important in the geological record and up to now considered an exclusively marine group of species^33^ (Fig. 6). Its marine genealogy places *P. cetinaensis* as a secondary FW immigrant^32^. The inability to live in the estuary of the Cetina River where there is at least some influence of diluted seawater, along with the development of sexual and asexual reproductive structures (conceptacles, Figs 2e,3d–f and 4), vouch for its full adaptation to FW conditions.

A scenario invoked to explain many FW invasions is the landlocking of marine species as a result of sea-level changes at different spatial and temporal scales, with the subsequent dilution of seawater. Most marine species would vanish in such new conditions, and only on very rare occasions would they adapt to the new environment^34^. The most serious obstacles preventing invasion into FW are the regulation of osmotic pressure, ionic concentration, pH level, low temperature, constant runoff, food resources, competition, and available living space^26^,^34^. All of these hurdles were surmounted by *P. cetinaensis* in recent geological history, probably due to the preadaptation of its brackish-water ancestor and the specific characteristics of the karst Cetina River.

About 120,000 years ago, global sea levels began descending from their maximum level, which was slightly higher than present-day levels^35^, to a minimum 20,000 years ago, which corresponds to the last glacial maximum when sea levels were about 120 m below present-day levels^36^. This was not a continuous drawdown, as there were many reversals in the descending trend^37^. During the last glacial maximum, the Cetina River had to cross four depressions (today at 60 to 90 m below sea level) before reaching the Adriatic Sea^38^ (Fig. 7). In periods when the depressions were part of the Cetina River estuary and global sea level was descending or ascending, the depressions were probably inhabited by brackish species.

*Pneophyllum* spp. were typical of these brackish inhabitants, as they are today in the Adriatic lagoons, estuaries, and deltas where they flourish as inconspicuous epiphytes on seagrass^39^. As an inhabitant of a paleo-estuary, the ancestor of *P. cetinaensis* was preadapted to osmotic stress and rapid changes in water salinity and ambient temperature that rapidly oscillated beyond the thresholds of typical shallow-marine habitats. A small enhancement in osmotic regulation^40^, together with the common r-strategist and opportunistic nature of *Pneophyllum* spp., would result in offspring with higher fitness, equipped for full FW colonization. Specimens may have survived on the shallowest estuary bottom (mainly occupied by FW), and subsequently spread upstream. However, survival and spreading of *Pneophyllum* could not happen in just any type of river; rather, the specific characteristics of the karst Cetina River may have largely determined the favourable outcome.

For proper growth and cell wall calcification, *Pneophyllum*, like other coralline algae, requires calcium carbonate and magnesium, which are sufficiently present in seawater, but not in every type of river. As most of the Cetina River catchment lies on carbonate rocks, mainly limestone^19^, its water is hard and enriched in dissolved calcium carbonate and magnesium ions (Table 1), essential for the development of corallines. Moreover, high levels of ions in the Cetina River indicated by high conductivity (Table 1), made osmoregulation easier for marine species. Difficulties in osmoregulation during the transition from MW to soft water in compared with hard water water have been observed in the brackish water flatworm^41^, while alleviation of osmotic stress thanks to high concentrations of ions has been suggested as important for the establishment of a FW population of the primarily marine red alga *Polysiphonia subtilissima* Montagne^42^.

The calcified tissue of corallines cannot develop in an acidic environment^43^, such as in soft-water rivers or acid tropical rivers with dissolved fulvic and humic substances^44^. In particular a significant reduction in epiphytic coralline algal cover with increasing acidification due to natural CO_2_ vents has been reported by^45^. Therefore, the hard water of karst, carbonate rivers with pH values similar to the marine environment, as is the case of the Cetina River (median pH of 8.12), are the only potential ones for coralline algae invasion.

Unlike most FW invaders, *P. cetinaensis* did not encounter competitors and predators in the Cetina River^21^. Cobbles and pebbles in the Cetina River bed are mostly uncolonized by other macroalgae and mosses, which was probably also the case during the early invasion of *P. cetinaensis*. This substratum therefore provided a favourable, vacant habitat for the alga to occupy. Calcified cell walls give coralline algae excellent protection from herbivory, which promoted the diversification of specialized grazers in the sea^46^. The absence of specialized grazers favoured the establishment of *P. cetinaensis* in the Cetina River, where common river herbivores, mainly gastropods, amphipods, and insects^47^ cannot feed on calcified crusts. However, by feeding on epiphytes overgrowing *P. cetinaensis*, those herbivores probably perform the same beneficiary function as marine herbivores: cleaning coralline algal surfaces of fast-growing epiphytic species^48^.

Our observation suggests that FW gastropods, especially *T. fluviatilis fluviatilis,* have one additional, peculiar function in the biology of *P. cetinaensis*. This alga, like other red algae, does not have a vagile life stage and is constantly facing washout in the river stream. As most adult gastropods (more than 95% in the type locality) are overgrown by crusts of *P. cetinaensis*, commonly with developed reproductive organs (Fig. 2e), they serve as the main dispersal vector of *P. cetinaensis* through the river. In areas with no algal encrustation on pebbles, such as the slightly eutrophic part of the river in the vicinity of the town of Trilj (Fig. 1a), the algae can be found on gastropods, indicating an affinity of spores to attach onto gastropod shells and/or gastropod mobility. Most gastropod species can actively move upstream from 0.3 to 1.0 km per year^49^. Over periods of tens to hundreds of years, benthic molluscs could have dispersed *P. cetinaensis* over a distance of 75 km along the river, even surmounting waterfalls (up to 50 m high). Such pronounced malacochory is also benefited by one more peculiarity of karst rivers: a high predominance of gastropods among benthic macroinvertebrates^50^. In the area of the type locality (Fig. 1), gastropods represent around 40% of the total number of benthic macroinvertebrates, with a maximum density of almost 4,500 specimens m^−2^ 47.

The fact that other karst rivers close to the Cetina River share similar FW gastropod fauna^50^, but lack *P. cetinaensis* despite the river’s proximity and pronounced transportation by gastropods, supports our hypothesis of a geologically recent biome transition and estimated onset of the Cetina River invasion within the last 120,000 years.

*Pneophyllum* is a widely distributed genus encompassing 18 species currently accepted taxonomically^16^. Seven species have been reported from the European Atlantic and Mediterranean coasts. All these taxa are represented in our molecular analyses by DNA sequences from type material (*P. lobescens, P. limitatum*, and *P. subplanum*) or from historical collections available for molecular studies. The exception is *P. zonale* (a species described from the Atlantic French coast growing on a small piece of glass^51^), for which all attempts to obtain molecular data from the isolectotype failed. The use of type specimens as taxonomical references confirms that the novel species described herein has not been formally described among European *Pneophyllum*; furthermore, it highlights the presence of additional cryptic species within the genus. It is obvious that further systematic research is required to assess the diversity within the genus *Pneophyllum*, for which current taxonomical features for species determination might be insufficient.

**Figure 5:**
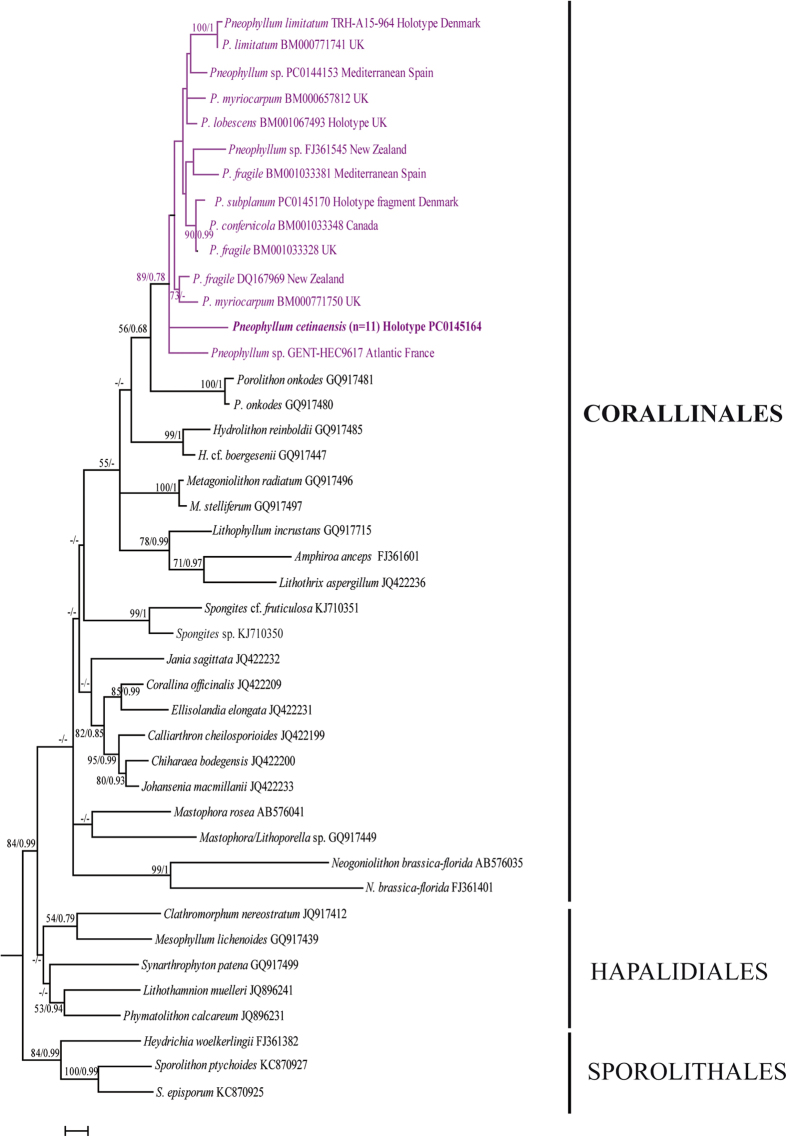
Phylogenetic tree inferred from ML and BI analyses of the *psb*A sequences of *Pneophyllum cetinaensis, Pneophyllum* taxa recorded in the Atlantic Ocean and Mediterranean Sea (*P. zonale, P. subplanum, P. limitatum* are represented by their type collections), and genera from the orders Corallinales, Hapalidiales, and Sporolithales. Boostrap ML values >50% and posterior probabilities >0.50 from Bayesian inference are shown for each node. Members of the order Sporolithales were used as outgroup. Scale bar: 0.02 substitutions per site.

**Figure 6:**
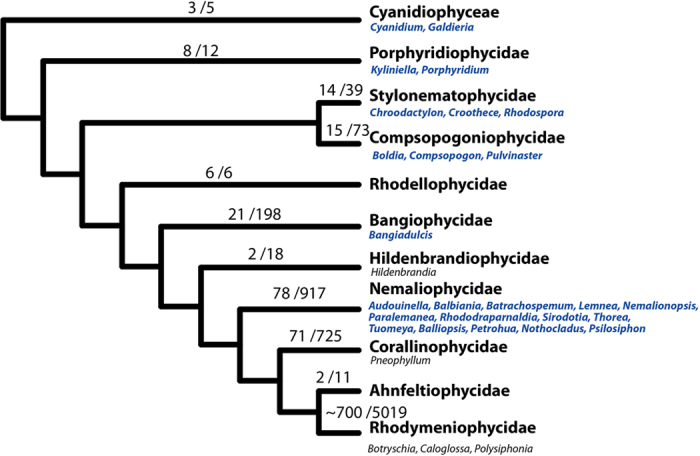
Phylogenetic relationships of red algae. Branches showing the currently number of genera and species estimated from AlgaeBase^16^. FW genera are listed under the leaf showing the major lineages among the Rhodophyta. Genera in blue include exclusively FW species.

**Figure 7:**
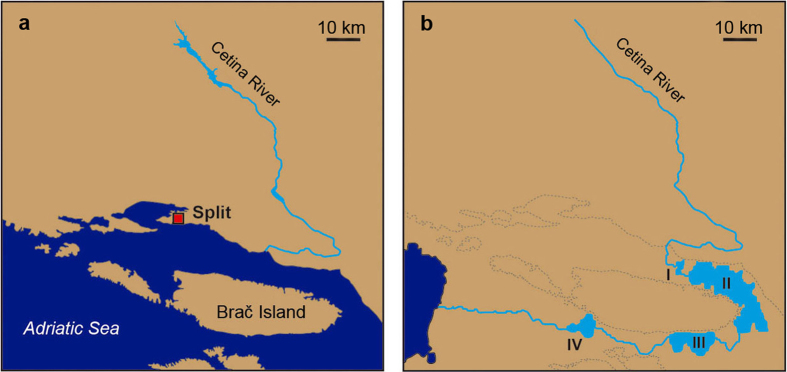
The Adriatic coast in the area of the Cetina River mouth. Recent situation (**a**) and the Cetina River about 20,000 years ago (**b**) with sea level at −115 m. The Cetina River ran through four paleolakes (I to IV), filling the depressions before reaching the Adriatic Sea. Under a scenario of sea-level oscillations, these paleo-lakes repeatedly became part of a paleo-estuary, most likely facilitating adaptation to FW conditions and peripatric speciation of the ancestor of *Pneophyllum cetinaensis*. Grey lines indicate the present-day shoreline. Maps were created using Adobe In Design CS5 and Photoshop CS5 software and based on OpenStreetMap (https://www.openstreetmap.org/copyright). Cetina paleo-course is drawn based on the data from^38^.

The molecular data places *P. cetinaensis* in the *Pneophyllum* clade, and also negates close phylogenetic relation to any other recognized European species (Fig. 5). The results suggest many intriguing questions for future studies: what happened with the marine/brackish water ancestor of *P. cetinaensis*? Does the ancestral species still have marine/brackish water descendants or have they vanished?

Taking into account our present insufficient knowledge of the diversity of the *Pneophyllum* clade and that *P. cetinaensis* does not have close relatives among described marine *Pneophyllum* species, we can suppose that a marine/brackish species closely related to *P. cetinaensis* might still be found in the Adriatic/Mediterranean Area.

*Pneophyllum cetinaensis* defies the paradigm of coralline algae being exclusively marine species. The fact that coralline algae can exist in a FW habitat will probably open new discussions and produce significant impacts in different fields. Standard textbook concepts consider coralline algae as paleoenvironmental indicators, stating that they are “commonly adapted to normal marine salinities”^52^, although it is acknowledged that they can tolerate brackish to hypersaline conditions^53^.

Changes in sea levels throughout the Pleistocene resulted in several well-documented peripatric populations of marine species isolated in marine lakes, which serve as a suitable subject for the research of evolutionary processes^54^. *Pneophyllum cetinaensis* also has a large potential to become a model organism to study evolution through peripatric speciation. Furthermore, as a species that crossed the border between the marine and freshwater biomes, it is of particular interest for studying the ecophysiological mechanisms and underlying genomic characteristics of the transition. The Cetina River is in a karst region of the Balkan Peninsula that is unique due to numerous endemic species in FW, sea, land, and especially in caves. During the Pleistocene glaciation, this area (along with the Iberian Peninsula and the Apennines) was a major refugium for European species as it remained largely unaffected by glaciers. Consequently, many species survived glaciations, and due to the karst geology, they remained isolated and evolved as endemic species^55^. The Adriatic basin has more than 40 endemic FW fish species, many endemic to only one river (sometimes very short). In addition, there is also a notable quantity of species that would never be expected to occupy the habitat in which they are found. Examples of such unexpected species in the Balkan area are the only known cave sponge *Eunapius subterraneus*, the rare stygobitic cave leech *Croatobranchus mestrovi*, the unique FW cave-dwelling tube worm *Marifugia cavatica*, the only underground bivalve in the world *Congeria kusceri*, and the only cave-dwelling chordate species found in Europe *Proteus anguinus*^56^. *Pneophyllum cetinaensis*, a unique FW coralline alga, takes its place among these species, confirming that the Balkan Peninsula is a hot spot for endemism and peculiar species, many probably yet to be discovered. Although finding a new species today is not unusual, the discovery of a FW coralline alga is quite surprising.

## Methods

### Field observations and sampling

Following our initial observation of *Pneophyllum cetinaensis* in 2013, we inspected the Cetina River at numerous locations from the river mouth to the river spring, including tributaries (Fig. 1). The aim was to collect data on distribution, as well as biotic and abiotic elements that might serve to characterize the biology and ecology of the species. Samples were collected for morphological and molecular analyses on the type locality (Fig. 1, Supplementary Table S1). Data on physical and chemical parameters were obtained from Hrvatske vode (the legal entity for water management in Croatia). The dataset includes measurements from the start of 2009 till the end of 2013 at two gauging stations covering the lower (Radmanove mlinice station) and upper (Cetina station) river courses (Fig. 1). Sampling was basically made on monthly interval. Radmanove mlinice station is in the type locality of *P. cetinaensis*.

We studied the frequency of algal development on gastropods by sampling numerous snail specimens from randomly collected cobbles and pebbles in the type locality (Island of Love) (Fig. 1). The presence and abundance of algal crusts was assessed under stereo-microscope taking into account gastropod species, size (larger or smaller than 4 mm), and coverage by the alga (under or above 50% of the shell surface). Two nearby karst rivers, the Jadro and Žrnovnica, were checked thoroughly for possible alga occurrences (Fig. 1).

### Morphological analyses

Specimens were air dried and stored in silica gel. Fragments were mounted on aluminium stubs and coated with gold/palladium (with S150 Sputter Coater, Edwards, Crawley, UK) prior to viewing with a LEICA Steroscan 430i (Cambridge, UK) at 20 kV. For the study of the reproductive cycle, fresh samples were collected and stored in 10 L dark plastic containers and transported to the laboratory within 12 hours, along with FW stored in several 25 L containers. The culture was set up in a thermoconstant room at 14 °C; five marked microscopy slides were placed at the bottom of the aquarium for the settlement of spores.

### Molecular study

We studied 11 specimens of *P. cetinaensis,* recent collections of *Pneophyllum* taxa in the Mediterranean and Atlantic Europe, as well as type species (*P. fragile, P. lobescens, P. limitatum, P. subplanum, P. zonale*) and other important historical collections of *Pneophyllum* species collected over the last two centuries and deposited at the Natural History Museum (BM), the Muséum National d´Histoire Naturelle (PC), and at the Norwegian University of Science and Technology (TRH) (see Supplementary Table S1 online). Except for *P. cetinaensis,* the rest of the collections were collected in marine areas, intertidally or subtidally, growing as epiliths on stones or glass but also as epiphytes on seaweeds and seagrasses (see Supplementary Table S1 online).

*DNA extraction, PCR, and PCR product sequencing.* Specimen surfaces without epiphytes were selected under a stereomicroscope and ground with a 2 mm drill bit for DNA extraction. Genomic DNA was extracted using a NucleoSpin® 96 Tissue kit (Macherey-Nagel, GmbH and Co. KG, Germany) following the manufacturer’s protocol. For *P. cetinaensis*, type specimens, and historical collections, we employed the QIAamp® DNA Micro Kit (Qiagen S.A.S., France) following the manufacturer’s protocol for tissues. The plastid gene encoding the D1 protein of photosystem II (*psb*A) was amplified in one reaction using the pairs of primers *psb*A-F1/*psb*A-R2 or *psb*A-F1/*psb*A600R^57^ following the thermal profile^58^. The PCR reaction mixture followed^59^, except for the amplification of type specimens and historical collections for which the DNA template was not diluted. PCR products were purified and sequenced by Eurofins (Eurofins Scientific, France). Voucher specimens for *P. cetinaensis* and recent collections of *Pneophyllum* were deposited in the Muséum National d´Histoire Naturelle (PC), Natural History Museum Split (NHMS), Herbarium Croaticum - University of Zagreb (ZA), and the Croatian Natural History Museum (CNHM)^60^. Sequences were submitted to the Barcode of Life Data Systems (project “NGCOR”, BOLD, http://www.boldsystems.org and GenBank (accession numbers listed in Supplementary Table S1 online). For the molecular analyses, publicly available sequences of *Pneophyllum* were included, as well as sequences from other genera of the orders Corallinales, Hapalidiales, and Sporolithales (see Supplementary Table S1 online).

*Molecular analyses.* Models of sequence evolution were estimated using the Akaike Information Criterion (AIC) and the Bayesian Information Criterion (BIC) obtained in jModeltest 2.1.3^61^. Maximum Likelihood analysis for the *psb*A alignment was performed under a generalized time-reversible with gamma+invariant sites heterogeneity model (GTR+G+I), and the bootstrap consisted of 1,000 replicates. The *psb*A alignment comprised 44 haplotype sequences ranging from 376 to 851 bp, with 294 variable sites. The alignment did not include either the holotype fragment of *P. fragile* or the isolectotype of *P. zonale*, for which DNA sequences could not be obtained.

## Additional Information

**How to cite this article**: Žuljević, A. *et al.* First freshwater coralline alga and the role of local features in a major biome transition. *Sci. Rep.* **6**, 19642; doi: 10.1038/srep19642 (2016).

## Supplementary Material

Supplementary Dataset 1

## Figures and Tables

**Table 1:** Physichochemical parameters of upper and lower courses of Cetina River.

Parameter	Range	Average ± s.d. (μ) | Median (M)
water temperature (°C)		
upper course	4.8–18.7	10.4 ± 3.0 (μ)
lower course	6.1–18.9	12.5 ± 3.4 (μ)
pH		
upper course	7.7–8.3	8.1 (M)
lower course	7.8–8.3	8.2 (M)
total hardness (mg CaCO_2_ l^−1^)		
upper course	176–237	200 ± 16 (μ)
lower course	180–243	203 ± 12(μ)
calcium hardness (mg CaCO_2_ l^−1^)		
upper course	138–201	166 ± 14 (μ)
lower course	116–218	171 ± 15 (μ)
conductivity (μS cm^−1^)		
upper course	282–602	407 ± 90 (μ)
lower course	305–473	372 ± 49 (μ)

Notes: Sampling stations are indicated in Fig. 1a. The type locality of *Pneophyllum cetinaensis* is in the lower course. Number of data collected through 5 years period: upper course n = 36, lower course n = 42. s.d.: standard deviation.
