# Supplementary material for: First freshwater coralline alga and the role of local features in a major biome transition
Source: Sci Rep. 2016 Jan 21;6:19642. doi: 10.1038/srep19642 (PMC4726424; doi:10.1038/srep19642)
Supplement: Supplementary Dataset 1 [file srep19642-s1.doc]

**SUPPLEMENTARY INFORMATION**

**First freshwater coralline alga and the role of local features in a major biome transition**

A. Žuljević, S. Kaleb, V. Peña, M. Despalatović, I. Cvitković, O. De Clerck, L. Le Gall, A. Falace, F. Vita, Juan C. Braga, and B. Antolić

**Supplementary Table 1: Sample information for *Pneophyllum* species included in the molecular analyses.** In bold letters, the collection details and type material of *Pneophyllum cetinaensis.* Additional sequences available from GenBank are also detailed.

| **Order** | **Family** | **Species** | **Collection details** | **GenBank Accession number** | **Herbarium** |
| --- | --- | --- | --- | --- | --- |
| **Corallinales** | **Corallinaceae** | ***Pneophyllum cetinaensis* sp. nov.** | **Otok Ljubavi, Cetina river, Croatia (43° 26.180'N - 16° 45.785'E). 27/ 08/2013. Coll: Žuljević, A. Det: Kaleb, S.** |  | **Holotype:** |
|  |  |  |  | **KT783425** | **PC0145164** |
|  |  |  |  |  | **Isotypes:** |
|  |  |  |  | **KT783415** | **PC0145167** |
|  |  |  |  | **KT783424** | **PC0145166** |
|  |  |  |  | **KT783429** | **PC0145165** |
|  |  |  |  | **KT783420** | **NHMS000566** |
|  |  |  |  | **KT783421** | **NHMS000567** |
|  |  |  |  | **KT783433** | **NHMS000568** |
|  |  |  |  | **KT783431** | **CNHM600:ZAG;1:BOA** |
|  |  |  |  | **KT783432** | **ZA39846** |
|  |  |  |  | **KT783422** | **ZA39848** |
| Corallinales | Corallinaceae | *P. subplanum* (Rosenvinge) Y.M.Chamberlain ex South & Tittley | Holotype fragment. On seagrass. Horsens, Samsø, Denmark. 17/09/1893. Coll: Rosenvinge, L.K. | KT783430 | PC0145170 |
| Corallinales | Corallinaceae | *P. lobescens* Y.M. Chamberlain | Holotype. Subtidal (-8 m), on glass. Mouth of the River Yealm, South Devon, United Kingdom. 22/10/1977. Coll: Gray, P.W.G. & Critchley, A.T. | KT783416 | BM001067493 |
| Corallinales | Corallinaceae | *P. limitatum* L.K. Rosenvinge | Holotype. On *Fucus vesiculosus* Linnaeus. Limfjorden, Jylland, Denmark. 22/08/1893. Coll: Rosenvinge, L.K. | KT783423 | TRH- A15-964 |
| Corallinales | Corallinaceae | *P. limitatum* | Intertidal, on *Palmaria palmata* (Linnaeus) Weber & Mohr. Bembridge, Isle of Wight, United Kingdom. 24/09/1980. Coll: Chamberlain, Y.M. | KT783427 | BM000771741 |
| Corallinales | Corallinaceae | *P. myriocarpum* (P.Crouan & H.Crouan) Y.M.Chamberlain | Yealm River, South Devon, United Kingdom. 22/10/1977. Coll: Gray,P.W. & Critchley,A.T. | KT783418 | BM000771750 |
| Corallinales | Corallinaceae | *P. myriocarpum* | Langerstone Point, South Devon, United Kingdom. 04/03/1984. Coll: Chamberlain, Y.M. | KT783413 | BM000657812 |
| Corallinales | Corallinaceae | *P. fragile* Kützing | Subtidal, on Posidonia Oceanica. Almadra, Spain. 09/08/1981. Coll: Chamberlain, Y.M. | KT783426 | BM001033381 |
| Corallinales | Corallinaceae | *P. fragile* | Intertidal, on *Zostera marina* Linnnaeus. Isle of Wight, United Kingdom. 24/09/1980. Coll: Chamberlain, Y.M. | KT783417 | BM001033328 |
| Corallinales | Corallinaceae | *P. confervicola* (Kützing) Y.M. Chamberlain | Intertidal, on *Zostera marina*. St-Paul´s Inlet, Canada. 18/10/1972. Coll: R. Hooper. | KT783434 | BM001033348 |
| Corallinales | Corallinaceae | *Pneophyllum* sp. | Intertidal, on *Gelidium spinosum* (S.G. Gmelin) P.C.Silva. Atlantic coast of France. 05/05/1993. Coll: Coppejans, E. | KT783414 | GENT-HEC9617 |
| Corallinales | Corallinaceae | *Pneophyllum* sp. | Subtidal (-15 m). La Herradura, Granada, Mediterranean Spain. 19/09/2012. Coll: Le Gall, L. & Peña, V. | KT783428 | PC0144153 |
| **Additional sequences data included in the alignment** | | | | | |
| Corallinales | Corallinaceae | *Amphiroa anceps* (Lamarck) Decaisne | New Zealand^1^ | FJ361601 |  |
| Corallinales | Corallinaceae | *Calliarthron cheilosporioides*Manza | Canada^2^ | JQ422199 |  |
| Corallinales | Corallinaceae | *Chiharaea bodegensis* H.W. Johansen | Canada^2^ | JQ422200 |  |
| Corallinales | Corallinaceae | *Corallina officinalis* Linnaeus | Canada^2^ | JQ422209 |  |
| Corallinales | Corallinaceae | *Ellisolandia elongata* (Ellis & Solander) K. Hind & G.W. Saunders | Ireland^2^ | JQ422231 |  |
| Corallinales | Corallinaceae | *Hydrolithon reinboldii* (Weber-van Bosse & Foslie) Foslie | New Caledonia^3^ | GQ917485 |  |
| Corallinales | Corallinaceae | *H.* cf. *boergesenii* (Foslie) Foslie | Vanuatu (as "Uncultured Corallinales")^3^ | GQ917447 |  |
| Corallinales | Corallinaceae | *Jania sagittata* (J.V. Lamouroux) Blainville | Australia^2^ | JQ422232 |  |
| Corallinales | Corallinaceae | *Johansenia macmillanii* (Yendo) K. Hind & G.W. Saunders | Canada^2^ | JQ422233 |  |
| Corallinales | Corallinaceae | *Lithophyllum incrustans* Philippi | Spain (as "Uncultured Corallinales")^3^ | GQ917715 |  |
| Corallinales | Corallinaceae | *Lithothrix aspergillum* J.E. Gray | Canada^2^ | JQ422236 |  |
| Corallinales | Corallinaceae | *Mastophora rosea* (C. Agardh) Setchell | Japan^4^ | AB576041 |  |
| Corallinales | Corallinaceae | *Mastophora/Lithoporella* sp. | Vanuatu (as "Uncultured Corallinales")^3^ | GQ917449 |  |
| Corallinales | Corallinaceae | *Metagoniolithon radiatum* (Lamarck) Ducker | Australia (as "Uncultured Corallinales")^5^ | GQ917496 |  |
| Corallinales | Corallinaceae | *M. stelliferum* (Lamarck) Ducker | Australia (as "Uncultured Corallinales")^5^ | GQ917497 |  |
| Corallinales | Corallinaceae | *Neogoniolithon brassica-florida* (Harvey) Setchell & L.R.Mason | Japan^4^ | AB576035 |  |
| Corallinales | Corallinaceae | *N. brassica-florida* | New Zealand, Broom *et al.* (unpublished) | FJ361401 |  |
| Corallinales | Corallinaceae | *Pneophyllum fragile* | New Zealand, Hart *et al.* (unpublished) | DQ167969 |  |
| Corallinales | Corallinaceae | *Pneophyllum sp.* | New Zealand^1^ | FJ361545 |  |
| Corallinales | Corallinaceae | *Porolithon onkodes* (Heydrich) Foslie | New Caledonia^3^ | GQ917481 |  |
| Corallinales | Corallinaceae | *P. onkodes* | New Caledonia (as "Uncultured Corallinales")^3^ | GQ917480 |  |
| Corallinales | Corallinaceae | *Spongites* cf. *fruticulosa* Kützing | Greece^6^ | KJ710351 |  |
| Corallinales | Corallinaceae | *Spongites* sp. | Guadeloupe, French West Indies^6^ | KJ710350 |  |
| Hapalidiales | Hapalidiaceae | *Clathromorphum nereostratum* Lebednik | not available, Maneveldt *et al.* (unpublished) | JQ917412 |  |
| Hapalidiales | Hapalidiaceae | *Lithothamnion muelleri* Lenormand ex Rosanoff | Mexico^7^ | JQ896241 |  |
| Hapalidiales | Hapalidiaceae | *Mesophyllum lichenoides* (Ellis) Me. Lemoine | Spain^3^ | GQ917439 |  |
| Hapalidiales | Hapalidiaceae | *Phymatolithon calcareum* (Pallas) W.H. Adey & D.L. McKibbin | England^7^ | JQ896231 |  |
| Hapalidiales | Hapalidiaceae | *Synarthrophyton patena*  (J.D. Hooker & Harvey) R.A. Townsend | Australia (as "Uncultured Corallinales")^5^ | GQ917499 |  |
| Sporolithales | Sporolithaceae | *Heydrichia woelkerlingii* R.A. Townsend, Y.M. Chamberlain & Keats | New Zealand^1^ | FJ361382 |  |
| Sporolithales | Sporolithaceae | *Sporolithon episporum* (M.A. Howe) E.Y. Dawson | Costa Rica^8^ | KC870925 |  |
| Sporolithales | Sporolithaceae | *S. ptychoides* Heydrich | Brazil^8^ | KC870927 |  |

**Supplementary References:**

1. Farr, T., Broom, J., Hart, D., Neill, K. & Nelson,W. Common coralline algae of northern New Zealand. An identification guide., Science communicator, NIWA edition, Wellington. 125 p (2009).

2. Hind, K. R. & Saunders, G. W. A molecular phylogenetic study of the tribe Corallineae (Corallinales, Rhodophyta) with an assessment of genus-level taxonomic features and descriptions of novel genera. *J. Phycol.* **49**, 103-114 (2013).

3. Bittner, L., Halary, S., Payri, C., Cruaud, C., De Reviers, B., Lopez, P. & Bapteste, E. Some considerations for analyzing biodiversity using integrative metagenomics and gene networks. *Biol. Direct* **5**, 47 (2010).

4. Kato, A., Baba, M. & Suda, S. Revision of the Mastophoroideae (Corallinales, Rhodophyta) and polyphyly in nongeniculate species widely distributed on Pacific coral reefs. *J. Phycol.* **47**, 662-672 (2011).

5. Bittner, L., Payri, C., Maneveldt, G., Couloux, A., Cruaud, C., De Reviers, B. & Le Gall, L. Evolutionary history of the Corallinales (Corallinophycidae, Rhodophyta) inferred from nuclear, plastidial and mitochondrial genomes. *Mol. Phylogen. Evol.* **61**, 697-713 (2011).

6. Peña, V., Rousseau, F., De Reviers, B. & Le Gall, L. First assessment of the diversity of coralline species forming maerl in Guadeloupe, Caribbean using an integrative systematic approach. *Phytotaxa* **190**, 190-215 (2014).

7. Hernández-Kantún, J.J., Riosmena-Rodriguez, R., Hall-Spencer, J.M., Peña, V., Maggs, C.A. & Rindi, F. Phylogenetic analysis of rhodolith formation in the Corallinales (Rhodophyta). *Eur. J. Phycol*. **50**, 46-61 (2015).

8. Bahía, R.G., Amado-Filho, G., Maneveldt, G., Adey, W.H., Johnson, G., Marins, B. V. & Longo, L.L. *Sporolithon tenue* sp. nov. (Sporolithales, Corallinophycidae, Rhodophyta): A new rhodolith-forming species from the tropical southwestern Atlantic. *Phycol. Res.* **62**, 44-54 (2014).
